# An RGS-Containing Sorting Nexin Controls *Drosophila* Lifespan

**DOI:** 10.1371/journal.pone.0002152

**Published:** 2008-05-14

**Authors:** Jae Myoung Suh, Drew Stenesen, John M. Peters, Akiko Inoue, Angela Cade, Jonathan M. Graff

**Affiliations:** 1 Department of Developmental Biology, University of Texas Southwestern Medical Center, Dallas, Texas, United States of America; 2 Department of Molecular Biology, University of Texas Southwestern Medical Center, Dallas, Texas, United States of America; 3 Department of Internal Medicine, University of Texas Southwestern Medical Center, Dallas, Texas, United States of America; University of California San Francisco, United States of America

## Abstract

The pursuit of eternal youth has existed for centuries and recent data indicate that fat-storing tissues control lifespan. In a *D. melanogaster* fat body insertional mutagenic enhancer trap screen designed to isolate genes that control longevity, we identified a regulator of G protein signaling (RGS) domain containing sorting nexin, termed *snazarus* (***s*** *orting* ***n*** *exin l* ***azarus***, *snz*). Flies with insertions into the 5′ UTR of *snz* live up to twice as long as controls. Transgenic expression of UAS-*Snz* from the *snz* Gal4 enhancer trap insertion, active in fat metabolic tissues, rescued lifespan extension. Further, the lifespan extension of *snz* mutants was independent of endosymbiont, e.g., *Wolbachia*, effects. Notably, old *snz* mutant flies remain active and fertile indicating that *snz* mutants have prolonged youthfulness, a goal of aging research. Since mammals have *snz*-related genes, it is possible that the functions of the *snz* family may be conserved to humans.

## Introduction

The interplay of several environmental variables such as predation, infection, and temperature, as well as availability of food and water are major regulators of lifespan [1]. Caloric restriction (CR) extends the lifespan of organisms from yeast to rodents, and characterization of its mechanisms is a major focus of longevity research [2]–[4]. Endosymbiotic infection has also been shown to influence *Drosophila* lifespan [5]. In addition to the environment, genes play an important role in lifespan control as highlighted by the striking interspecies variation of average lifespan (e.g., “dog years”). Genetic approaches, primarily in yeast, worms, and flies, have identified several key molecular components of longevity control [2], [6], [7]. Among these, Sir2, an important component of the CR pathway, the metabolic/oxidative state of the mitochondria, and reduced insulin/insulin-like growth factor signaling, appear to have conserved roles in invertebrate and mammalian longevity [8]–[10].

A few studies have been directed towards identifying tissue-specific control of longevity and some data show a role for the nervous system [11]. For example, transgenic expression of Sir2 in the fly brain can confer lifespan extension [11]. Sir2 interacts with and regulates the activity of PPARγ and PGC1, both of which play central roles in metabolism [12]. PPARγ is a master regulator of adipocyte biology, raising the possibility that Sir2 may also control lifespan through effects in metabolic and fat-storing tissues [13].

Food intake (CR) and insulin signaling also have important effects on metabolism and alter both adiposity and adipose function, indicating that fat-storing cells might be involved in longevity control [14], [15]. Many of the long-lived worm, fly, and mouse mutants have significant changes in adiposity, as do flies selected for postponed senescence [9], [16]–[19]. Flies store fat in the fat body, a central fly metabolic tissue, and fat body-restricted transgenic inhibition of insulin or TOR signaling increases *D. melanogaster* lifespan [20]–[22]. Remarkably, this effect is conserved to mice; adipocyte-restricted conditional deletion of the insulin receptor (FIRKO) extends murine longevity [9]. The long-lived FIRKO mice have reduced adiposity and altered adipose function, but normal caloric intake. Therefore, the accumulated data indicate that invertebrate and vertebrate fat-storing tissues play important roles in lifespan control.

Single gene mutations that extend *Drosophila* lifespan are relatively rare [17], [23]–[25]. In an attempt to identify new molecules and mechanisms that confer increased lifespan through fat metabolic tissues, we performed a two-component (Gal4; UAS-GFP) enhancer trap screen isolating 591 insertions that expressed GFP either specifically in the fat body, the fly adipose organ, or prominently in the fat body with secondary expression in other tissues that regulate fat storage and metabolism (e.g., oenocytes, anterior midgut) [26]–[28]. Next, we tested the lifespan of each enhancer trap line, selecting ten in which longevity was extended by >30% in both sexes. We focused on one line, C32, which had the greatest extension of lifespan of our long-lived strains. C32 flies contain an insertion into the 5′ UTR of *CG1514*. *CG1514* encodes a sorting nexin (Snx) that contains phospholipid binding and regulator of G protein signaling (RGS) domains that we termed *snazarus* (***s*** *orting* ***n*** *exin l* ***azarus***, *snz*, pronounced snaz). Snxs are involved in several aspects of endocytosis and regulate trafficking of signaling receptors [29], [30]. Several lines of evidence indicate that *snz* mutations lead to the observed increase in lifespan. For example, transgenic expression of *UAS-Snz* by the C32 enhancer trap GAL4, highly expressed in the fat body, rescued *snz* mutant longevity to normal. Further, two other independent *snz* alleles are long-lived, the three *snz* alleles display trans-heterozygous lifespan extension, and excisions of the C32 insertion revert lifespan towards control. Notably, old *snz* mutants remain physically active and fertile suggesting that the lifespan extension did not compromise these indicators of fitness. Mammals contain three Snz structural homologs-SNX13, SNX14, and SNX25-that, like Snz, contain a regulator of G-protein signaling (RGS) domain. SNX13, SNX14, and SNX25 are dynamically expressed in mammalian fat tissues, increasing with adipogenesis and obesity. Taken together, the data indicate that Snz, a member of a subgroup of Snxs expressed in invertebrate and vertebrate fat metabolic tissues, controls *Drosophila* longevity.

## Results

### Sequential Fat Body Enhancer Trap and Longevity Screen

**Figure 1:**
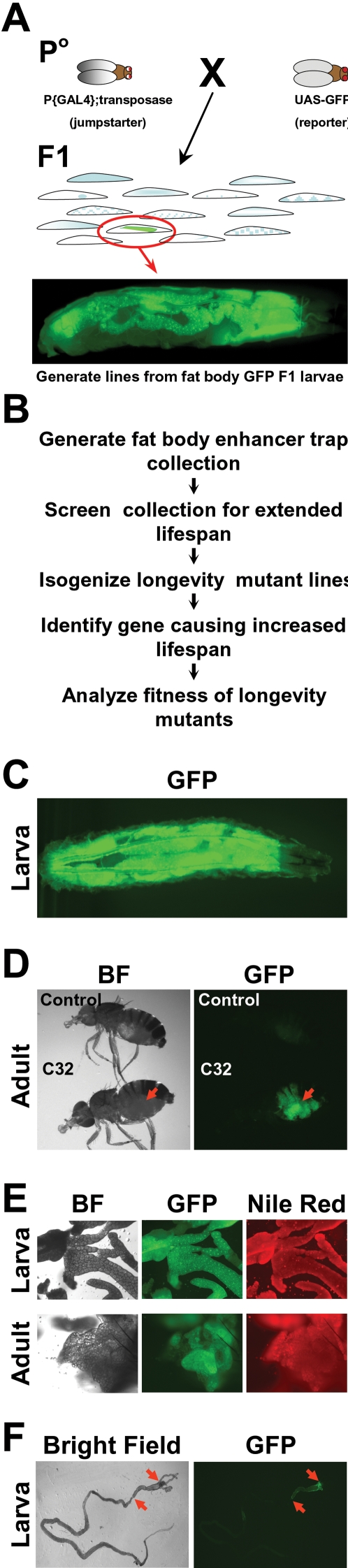
Fat Body Enhancer Trap Longevity Screen. (A) Cartoon of the two-component fat body enhancer trap screen. (B) Schema of the longevity screen. (C) *C32* (minimal promoter Gal4); *UAS-GFP* larvae were examined and photographed under GFP fluorescent microscopy, which showed strong expression in the fat body. (D) Adult control or *C32; UAS-GFP* flies were photographed under bright field (BF) or GFP fluorescent microscopy. Arrow indicates the abdomen, which contains much of the adult fat body. Slight background fluorescence in control fly is due to the presence of the yeast, which is used as a food source, in the gut. (E) The fat body of *C32; UAS-GFP* larvae or adults was removed, incubated with the fat specific fluorescent dye Nile Red and photographed under bright field (BF) as well as GFP and Nile Red fluorescent microscopy. (F) The intestinal tract of *C32; UAS-GFP* larvae were removed and then examined and photographed with GFP fluorescent microscopy, which showed that portions of the anterior midgut (segment between arrows) express GFP.

To identify regulators of lifespan that might function in tissues that control metabolism, we designed a sequential approach outlined in Figures 1A and 1B. The notion was to attempt to enrich for possible mutants through a fat body insertional enhancer trap screen followed by a comprehensive longevity screen. First, we performed a two-component (minimal promoter Gal4; UAS-GFP) F1 enhancer trap screen [31]; isolating lines with GFP expression in the fat body (Figure 1A). The presence of the minimal promoter-Gal4 in the mobilized P-element allowed us to not only generate mutants but also to simultaneously produce tools for fat-body transgenesis [31], [32]. We screened ∼700,000 F1 larvae, isolated 591 with fat body GFP expression, and generated lines from each. 102 of these lines displayed fat body-selective GFP expression, while others had some co-expression in other tissues that are critical in metabolism (i.e. fat body and oenocytes; fat body and anterior midgut; fat body, oenocyte, and anterior midgut) [26]–[28]. We also selected approximately 80 lines that did not have any GFP expression in the fat body but did express GFP in non-fat tissues such as brain or muscles; in part, to compare the frequency of possible long-lived flies in the two collections as a potential assessment of whether the regulation of lifespan might be biased towards one tissue or another.

Next we subjected the entire enhancer trap collection to repeated longevity studies in which we required reproducibly extended maximal lifespan, at a threshold of >30% increase compared to the mean, be present in both sexes and at both 25°C and 30°C. Since we assumed that lifespan extension at this stringency would be infrequent, and because in such a collection only ∼15% of the lines (i.e. ∼100) are projected to have any mutant phenotype [33], we initially screened the fat body enhancer trap lines in pools. Next, we sib selected the positive pools and screened individual lines several times. From this we identified ten lines that displayed a consistent and significant increase in lifespan. All of the lines that displayed increased longevity came from the fat body expressing enhancer trap lines and none derived from lines that we selected for lack of fat body expression, although this sample size was significantly smaller. We also compared the lifespan of the two longest lived lines to *mth* mutants, a previously described longevity mutant [24]. We found that in both males and females the lifespan extension conferred by these two lines approximated or slightly exceeded *mth* (not shown).

### The C32 Enhancer Trap Line Has Strong Larval and Adult Fat Body Expression

We further characterized the fat body enhancer trap line *C32*, whose insertion was located on the X chromosome, because it had the longest lifespan of the ten lines identified in the screen and because old *C32* flies appeared vigorous and fertile (see below). We examined the pattern of expression of *C32* by using the C32-Gal4 insertion to drive expression of UAS-GFP. We detected strong expression of GFP in the fat body from larval stages and throughout life (Figures 1C–1E). There was also some heterogeneous and lower level expression in the anterior midgut where fatty acid synthase and several other genes central to fat biology are expressed (Figure 1F, not shown) [34].

### The C32 Enhancer Trap Line Is Long-lived

**Figure 2:**
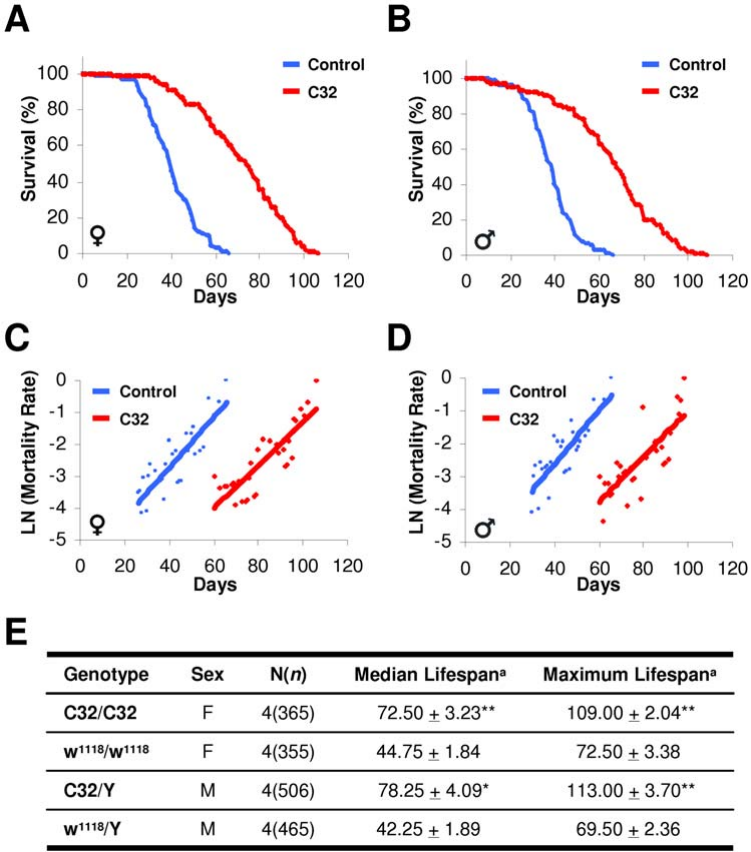
*C32* Flies Are Long-lived. (A, B) Adult female (A) or male (B) *C32* and control *w^1118^* flies were identically reared and survival was assessed daily. (n>80 per group, p<0.0001 by log-rank test) (C, D) Log mortality plots for adult female (C) or male (D) *C32* and control *w^1118^* flies identically reared. (n>80 per group, p<0.0001 by log-rank test) (E) Table summarizing four independent lifespan analyses of *C32* and control flies. ^a^Values are mean of the median and maximum lifespan of C32 and w^1118^ control female (F) and male (M) flies±standard error of the mean. N, number of replicates. n, total number of flies examined. * p <0.001, ** p <0.0003 by student's t-test.

To decrease the likelihood that second site mutations were the cause of the extended longevity, to avoid background or modifier effects, and to determine whether the increased lifespan was present in more than one genetic background, we backcrossed *C32* >10 generations into a control *w^1118^* strain. Then we studied *C32* hemizygous males and *C32* homozygous mutant females. We analyzed the lifespan of multiple cohorts of female and male *C32* mutants and sibling controls, finding that *C32* flies in the *w^1118^* background of either sex lived up to 100% longer than control *w^1118^* flies (Figures 2A-2E). Mortality analyses indicated that the primary effect of the *C32* mutation was a reduction of initial mortality rate (Figures 2C and 2D). These results show that the *C32* mutant allele leads to increased lifespan in both a mixed genetic background, i.e., the original enhancer trap mutant strain, and in the *w^1118^* inbred background.

### *C32* Inserted into the 5′ UTR of *snazarus*, which Encodes an RGS-containing Sorting Nexin

**Figure 3:**
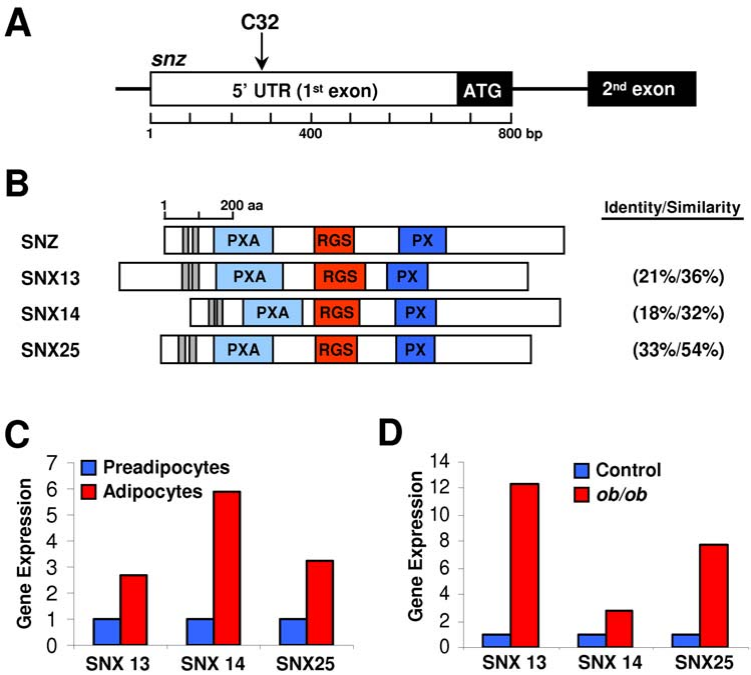
*C32* Enhancer Trap P-element Inserted into the 5′UTR of *snazarus.* (A) Location of the *C32* P-element insertion into the first exon of the *snazarus (CG1514)* gene. (B) Domain structure and alignment of *D. melanogaster* Snz and the three mammalian homologs. Grey rectangles represent hydrophobic patches (potential transmembrane domains). (C) SNX13, SNX14 and SNX25 mRNA expression levels were quantified with real-time PCR in uninduced 3T3-L1 preadipocytes and in induced 3T3-L1 adipocytes (n = 1). (D) Fat pads were removed from control and genetically obese *ob*/*ob* mice and the levels of SNX13, SNX14 and SNX25 expression were assessed with real-time PCR (n = 1).

To identify the genomic location of the *C32* P-element insertion, we sequenced DNA isolated from *C32* flies using plasmid rescue and inverse PCR at both the 5′ and 3′ ends–all these approaches identified only a single X chromosome insertion in cytogenetic band position 7C2-3. Database searches showed that the *C32* P-element inserted into the 5′UTR of *CG1514* (Figure 3A), a hypothetical gene that encodes a member of the sorting nexin (Snx) family, which we termed *snazarus* (*snz*) for ***s*** *orting* ***n*** *exin l* ***azarus***. Snx proteins are defined by the presence of a phospholipid binding (PX) domain (Figure 3B) [29], [30]. CG1514 also has two predicted transmembrane domains, a PX associated (PXA) domain, and a regulator of G-protein signaling (RGS) domain (Figure 3B). Database searches also identified three mammalian homologs of CG1514, termed SNX13, SNX14, and SNX25 (Figure 3B)[30]. We examined the expression of the three in 3T3-L1 preadipocytes, which can be induced to differentiate into adipocytes [35], and in fat depots from control and *ob*/*ob* mice, a genetic model of obesity [36]. We found dynamic expression of Snz homologs in 3T3-L1 cells and murine fat pads with higher levels in adipocytes (Figure 3C) and in *ob*/*ob* fat depots (Figure 3D).

### *Snz* Transgensis Rescues C32 Lifespan Extension

**Figure 4:**
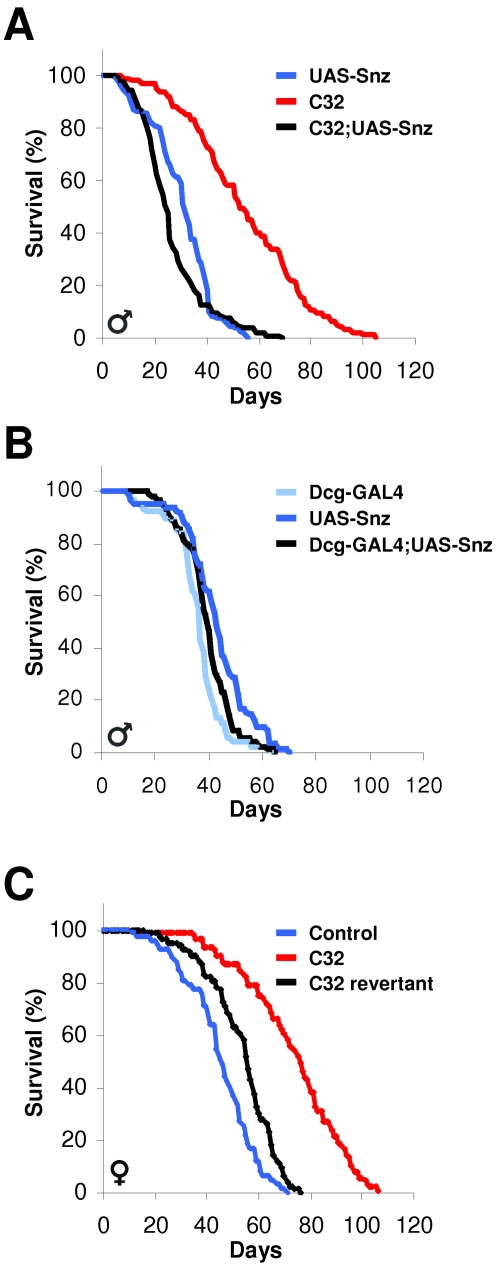
*Snz* Transgenesis and C*32* Excision Reduces Lifespan Extension. (A) The lifespan of male control, *C32*, and *C32; UAS-Snz* transgenic flies was plotted. (n>80, p<0.0001 by log-rank test between *C32* and *C32; UAS-Snz* ) (B) The lifespan of male *Dcg-GAL4*, UAS-Snz, Dcg-GAL4;UAS-Snz transgenic flies were plotted. (n>80) (C) Female control, *C32*, and *C32* revertant flies were cultured and survival was plotted. (n>80, p<0.0001 by log-rank test between *C32* and *C32* revertant) The lifespan of the male excision lines also reverted towards normal. Representative data from multiple experiments is shown.

To determine if *Snz* can rescue *C32* lifespan extension, we generated transgenic flies that contain full-length *Snz* cDNA under the control of an upstream activating sequence (UAS) that can be activated with Gal4, which was present in the P-element mobilized in our fat body enhancer trap screen. The *C32-Gal4* enhancer trap displayed strong expression in the larval and adult fat body and had some co-expression in the larval anterior midgut, a region that regulates fat storage (Figures 1C–1F) [28]. Since *C32* hemizygous males were long-lived, the presence of the Gal4 in the *C32* P-element allowed us to determine the lifespan effects of *Snz* transgenesis simply by crossing *UAS-Snz* males with *C32* mutant virgin females and then comparing lifespans of *C32*, *UAS-Snz*, and *C32;UAS-Snz* male siblings. The mortality curves indicated that *Snz* transgenesis, from the promoter active in metabolic tissues, rescued the *C32* lifespan extension (Figure 4A). However, UAS-Snz expressed from the Dcg-Gal4 fat body driver did not alter longevity (Figure 4B), which together with the relatively modest reduction in *C32;UAS-Snz* lifespan compared to control suggests that the reduction in longevity was neither non-specific nor due to Snz toxicity. The accumulated data support the notion that *Snz* regulates longevity and indicate that it does so in tissues central to fat biology.

### Excision of the C32 P-element Shortens Lifespan Extension

To further examine the possibility that *snz* is the gene that regulates lifespan in the *C32* line, we mobilized the *C32* P-element with an activated transposase and scored for excision of the P-element based upon an eye color marker [32]. We then evaluated progeny lines with altered eye color with a PCR-screening strategy designed to identify precise excision alleles. However, in even the most precise excision events, there still remained small alterations at the locus containing between 25 and 45 bases of the original P-element as is often observed [37]. We compared the lifespan of the three lines that most closely resemble the wild-type genomic sequence with *w^1118^* controls and the parent *C32* line and found that their longevity was more similar to control (Figures 4C, not shown).

### Independent Insertions into *snz* Have Increased Longevity and Display Transheterozygous Lifespan Extension

**Figure 5:**
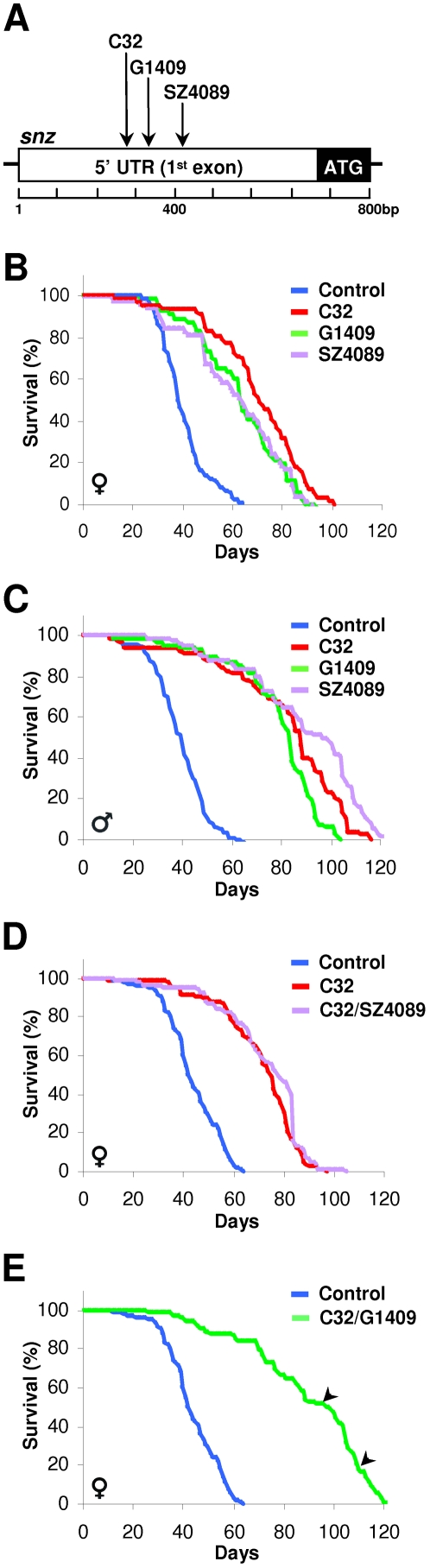
Independent *snz* Alleles Are Long-lived. (A) Location of the *C32*, *G1409*, and *SZ4089* P-element insertions in the *snazarus* locus. (B, C) Female (B) and male (C) control, *C32, G1409*, and *SZ4089* flies were cultured and survival was plotted. (n>80) (D, E) The lifespan of control, *C32* as well as *C32/SZ4089* (D) and *C32/G1409* (E) female transheterozygotes was assessed and plotted. (n>80) p<0.0001 by log-rank test between control and all *snz* mutant alleles. Arrowhead indicates fertility at observed timepoint. Representative data from multiple experiments is shown.

Several consortia have undertaken large-scale P-element insertional mutagenic screens [38]-[40]. To reduce the possibility that a linked second-site mutation was responsible for the *C32* lifespan extension, and to examine the generality of the effect, we identified and obtained two independently derived P-element *snz* insertions (*G1409* and *SZ4089*) that were both in the 5′UTR (Figure 5A). We then compared the lifespan of control, *C32*, *G1409*, and *SZ4089* flies and found that females and males of all three lines with an insertion into the *snz* locus lived substantially longer than controls (Figures 5B and 5C). Next, we performed complementation tests with females, the X-chromosome location of *snz* precluded this analysis in males, and observed lifespan extension in transheterozygotes of *C32* with either *G1409* or *SZ4089* (Figure 5D and 5E). Of note, very old *C32/G1409* transheterozygous females remained fertile and produced viable offspring (Figure 5E, arrowheads).

### *C32* Mutant Flies Are Active and Fertile

**Figure 6:**
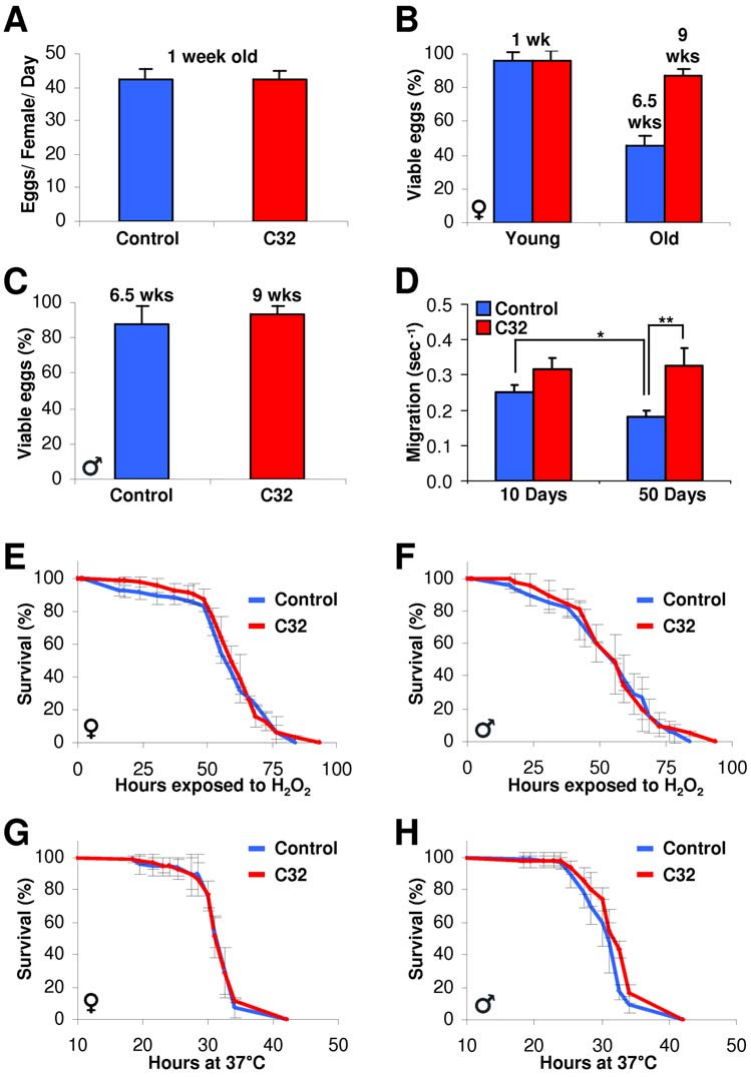
Old *C32* Flies Are Active and Fertile. (A) Egg production was measured for 1-week old control and *C32* females. (n = 15 per group) (B) Egg viability was assessed for control and *C32* females at the indicated ages. (n = 15 per group) (C) Male 6.5 week old controls and 9-week old *C32* flies were cultured with young control virgins and the percentage of viable eggs was evaluated. (n = 15 per group) (D) Activity, scored as crawling rate in a negative geotaxis assay, was analyzed in control and C32 flies at the indicated ages. (n = 30 per group) (E, F) 5 day-old adult *C32* and control flies, cultured in identical conditions, were incubated with H_2_O_2_ and female (E) and male (F) survival was plotted. (n = 60 per group) (G, H) Female (G) and male (H) control and *C32* flies were incubated at 37°C and survival was scored and plotted. (n = 60 per group) * p<0.02, ** p<0.03 by student's t-test. Error bars represent SEM.

The observation that very old *C32* flies, at ages when no control flies were alive, produced offspring was noteworthy as invertebrates and vertebrates have substantial reductions in fertility with aging and also because calorically restricted animals and many long-lived genetic mutants appear to shift from reproduction to somatic maintenance until conditions are again favorable for procreation [41], [42]. The extended fertility of the *C32* flies might reflect a delay in the onset of egg laying. So, we counted the number of eggs produced by 1 week-old control and 1week-old *C32* flies but found no difference (Figure 6A). Next, we analyzed the viability of eggs produced by 1-week old control and *C32* flies as well as cohorts at ∼50% survival (6.5-weeks old for controls versus 9-weeks old for *C32* mutants), a time point for *C32* flies at which virtually all control flies are dead (Figure 2). We found that at young ages the viability appeared indistinguishable (Figure 6B). However, the eggs produced by 6.5-week controls had substantially reduced viability while *C32* eggs were resistant to this effect (Figure 6B). Further, 9-week old *C32* flies produced approximately 4 times more progeny than 6.5-week controls (not shown). We also compared the ability of control and *C32* males at ∼50% survival to fertilize one-week old virgin control females and found that the percentage of viable eggs was equivalent (Figure 6C). Of note, development of *C32* flies proceeds normally based upon morphology and the timing of egg laying to eclosion.

In flies, reduced activity can extend lifespan and we wanted to assess whether *C32* may be such a “refrigerator” mutation [23]. Casual observations indicated that young and old *C32* flies were at least as, if not more, active than young controls in a variety of behaviors including feeding, flying, courting, and crawling. To quantify activity, we performed negative geotaxis assays [43], in which we analyzed the rate at which control and *C32* flies crawl from the bottom to the top of a vial. We found that *C32* flies were as, if not slightly more, vigorous as controls (Figure 6D), supporting the idea that the lifespan extension observed in *C32* flies is not secondary to decreased physical activity. Further, it appeared that that the *C32* mutation might ameliorate the decline in activity observed with aging (Figure 6D). Taken together, these data indicate that *C32* flies have an extension of not only lifespan, but also healthspan as assessed by fertility and activity.

The ability to handle environmental stress often decreases with age and many long-lived strains have improved stress responses, so we subjected *C32* and control flies to a variety of stressors [24], [25], [44], [45]. However in response to 5% H_2_O_2_, an oxidative stress, or elevated temperature, male and female *C32* flies had equivalent responses as sibling controls (Figures 6E–6H). These data suggest that *C32* flies have normal stress resistance.

### Endosymbiont Effects Do Not Account for *snz* Mutant Longevity

**Figure 7:**
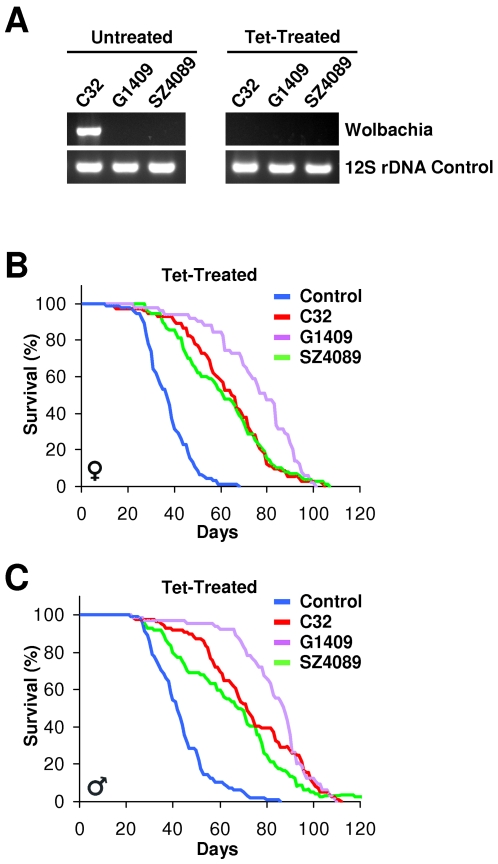
*Snz* Mutant Longevity Is independent of Endosymbiont Effects. (A) Detection of *Wolbachia* in PCR assay using *Wolbachia*-specific primers (upper panels) and universal bacterial 12S rDNA primers as a control (lower panels). Only the *C32* line was infected with *Wolbachia* (upper left panel) and infection was eliminated after tetracycline treatment (upper right panel). (B, C) The lifespan of female (B) and male (C) control and tetracycline-treated *C32*, *G1409*, and *SZ4089* flies are plotted. (n>80) p<0.0001 by log-rank test between control and all *snz* mutant alleles. Representative data from multiple experiments is shown.

Infections with endosymbionts, such as *Wolbachia*, can affect parameters of fitness such as fertility and lifespan and these effects can be reversed by treatment with the antibiotic tetracycline [5], [46], [47]. Since *snz* mutants had alterations that could result from such infections, we examined our *snz* mutant stocks for *Wolbachia* and found that one of the three *snz* mutant lines, *C32*, was infected based upon PCR genotyping with *Wolbachia* 16S rDNA-specific primers (Figure 7A)[48]. The absence of *Wolbachia* in the other two *snz* mutant lines indicated that this endosymbiont was likely not responsible for the *snz* mutant lifespan extension. However, other related infections or bacterial flora might contribute to the longevity phenotype. To address this possibility, we treated all lines with an extended course of tetracycline, which successfully eliminated *Wolbachia* from the *C32* line (Figure 7A). We then repeated the lifespan assays with the tetracycline-treated *snz* mutant lines, finding that the lifespan extension was maintained (Figures 7B and 7C). Thus, lifespan extension by *snz* mutant alleles appears independent of *Wolbachia* or other tetracycline-sensitive flora.

## Discussion

For centuries humans have searched for keys to long-life and recent experiments in a variety of model systems support the notion that fat tissues are important in longevity [9], [20]–[22]. To identify regulators of lifespan expressed in sites that regulate metabolism, we designed a multi-tiered approach, in which the initial step was a minimal promoter-Gal4; UAS-GFP fat body enhancer trap screen [31]. We analyzed the enhancer trap collection for lifespan, selecting ten lines that had significantly increased longevity during multiple assays in both males and females. These data indicate that fat body mutant collections are a rich resource to identify genes important in lifespan control. Our preliminary analyses indicate that our fat body enhancer trap screen did not approach saturation, so there are likely to be a diverse array of other genes that could be identified with related approaches directed towards metabolic tissues that could provide substantial insight into lifespan control. These could include additional F1 enhancer trap screens as well as other tiered longevity screens, for example first selecting for fat defects based upon buoyancy, starvation survival, or triglyceride content, might also be appropriate in the search for genes that regulate longevity.

We focused our attention on the *C32* line because these flies had the greatest extension of lifespan of any line in our collection and because old *C32* flies were active and fertile. *C32* inserted into the 5′UTR of the hypothetical gene *CG1514*, predicted to encode an RGS domain containing sorting nexin, that we termed *snazarus-* ***s*** *orting* ***n*** *exin l* ***azarus*** (*snz*, pronounced snaz). Several lines of evidence support the notion that *snz* is responsible for the longevity phenotype. For example, inverse PCR from both 5′ and 3′ ends of *C32* produce a single unique product and we only recovered one insertion in plasmid rescue experiments. Further, backcrossing the *C32* insertion >10 generations into a *w^1118^* background, to attempt to reduce both strain effects and second site mutations, maintained lifespan extension. To further examine the notion that *snz* was the responsible locus, we mobilized the *C32* P-element and found that the longevity of the most precise excision lines reverted towards control. However, these excision strains had longer life than control *w^1118^* flies, which may be secondary to background effects inherent in the methodology, the inability to backcross the revertants into *w^1118^* due to the loss of the eye-color marker, the presence of the remaining piece of the P-element, local hopping of the P-element into other regions of the *snz* gene, or other factors. Notably, two other independently derived P-element insertions in the *snz* locus, *G1409* and *SZ4089*, also conferred long life and display transheterozygous lifespan extension. These data indicate that the interval in which the P-elements are inserted confer the longevity phenotypes. However, there remains a possibility that *snz* is not the causative gene as the P-element insertions could alter linked genes or even have long-range effects, which could be clarified by identifying *snz* point mutations or with *snz* RNAi. We took an alternative approach and determined whether Snz transgenesis could regulate lifespan. We found that transgenic expression of Snz reverses the *C32* longevity phenotype, which indicates that Snz can alter lifespan. Thus the accumulated data are consistent with the idea that *Snz* regulates lifespan.

Snz is a member of the sorting nexin (Snx) family, defined by the presence of a PX, phospholipid binding domain [29], [30], [49]. A general theme of the Snx family is that they regulate various aspects of endocytosis, important in internalization and in modulating signal transduction [29], [30]. Many mammalian Snxs direct trafficking of surface receptors including tyrosine kinase receptors, in some cases increasing and in others reducing signal transduction [29], [30], [49]–[53]. Snz and the three related mammalian homologs, SNX13, SNX14, and SNX25, are a subgroup of the Snx family that, in addition to the signature PX domain, all contain an RGS domain indicating potential additional roles in signal transduction. RGS family proteins attenuate heterotrimeric G-protein signaling [54], [55]. The RGS domain of the Snz homolog SNX13 is unique among tested RGS domains in the ability to reduce signaling from Gαs proteins that regulate cAMP levels and thereby protein kinase A (PKA) action [49]. A recent study showed that activating the PKA pathway increased lifespan [56]. So, the RGS-containing Snx subgroup could control lifespan or metabolism by regulating protein trafficking and/or by modulating G protein signaling. Structure-function studies with Snz, such as attempting rescues with forms of Snz in which the PX or RGS domain is mutated, may help to clarify these notions.

Genetic studies have begun to identify mechanisms that regulate lifespan. These efforts have been hampered by the paucity of single gene mutants that display extended longevity. Recent data have raised the possibility that some of these few known mutants may not actually be long-lived [5]. Rather, the longevity appears induced by a complex interaction with an intracellular bacterium and the phenotype can be completely reversed by treatment with the antibiotic tetracycline [5]. Although this opens up the possibility to investigate genetic, environment, and flora interactions that may be important, they highlight the need to identify mutants that display longevity that is independent of bacterial contamination. Since the *snz* mutants have extended lifespan and enhanced fecundity, hallmarks of such infections, we treated the three *snz* alleles with the antibiotic tetracycline to evaluate possible dependence of the lifespan extension on the flora. However, even after the course of antibiotic therapy, all three *snz* mutants remained long-lived. Thus the mechanisms of lifespan extension conferred by reducing Snz action appear independent of tetracycline-sensitive microorganisms.

Studies of invertebrate mutants, and the responsible genes, have significantly contributed to our mechanistic understanding of lifespan control. Remarkably, many of the pathways that control invertebrate lifespan also appear likely to have related functions in mammals [2], [7], [9]. Many of these pathways are important in human health and especially in disorders of metabolism such as obesity and diabetes. For example, insulin signaling regulates invertebrate and vertebrate lifespan, and drugs that target this pathway are central diabetes therapies [7], [13], [57]. Further, Sir2 controls yeast, worm, and fly longevity and small molecules that target Sir2 improved mammalian metabolic parameters such as blood glucose levels [2], [8], [58], [59]. Here we describe a tiered, F1 strategy to identify flies with extended lifespan based upon enrichment for insertions in genes that are expressed in fat metabolic tissues. Given that relatively few single gene fly long-lived mutants have so far been identified, our data indicate that such collections are a rich resource to identify molecules important in lifespan control.

## Materials and Methods

### Fly Stocks and Culture

Flies were reared under uncrowded conditions in standard cornmeal-dextrose-agar-yeast media with sprinkled yeast granules unless otherwise noted. The original GAL4 enhancer trap insertion line, *pGawB/ FM7*, the transposase line *CyOHop2*, and the *w-; UAS-eGFP; UAS-eGFP* reporter line were previously described (gift from Dr. Claude Desplan) [31]. *w-; noc^Sco^/CyO; TM6B.Tb/MKRS* was from the Bloomington Stock Center (stock #3703). *w^1118^* and *mth^1^* (generous gifts from Dr. Seymour Benzer) were used as controls [24]. Dcg-GAL4 is a fat body GAL4 driver (gift from Dr. Charles Dearolf). Lines that were identified to have extended lifespan (see below) were backcrossed at least 10 generations into the *w^1118^* (control) background prior to further experiments. *G1409* (*P{Mae-UAS.6.11}CG1514,* gift from Dr. Ulrich Schafer) and *SZ4089* (*P{RS5}5-SZ-4089,* Szeged stock center) contain P-insertions into the *CG1514* locus. The precise location of P-insertion sites for all lines with lifespan extension were determined by inverse PCR (http://www.fruitfly.org/about/methods/inverse.pcr.html) and/or plasmid rescue. *C32* and *SZ4089* lines were backcrossed into the *w^1118^* (control) background by mating 20 mutant virgin flies, homozygous for the first cross and heterozygous for subsequent crosses, to 20 *w^1118^* males for 10 generations and then heterozygous females and hemizygous males were intercrossed to produce homozygous stocks. The P-element mutation was selected for by following the eye-color marker, mini-white. The *G1409* line does not have an eye color marker and thus was used in experiments without further backcrossing.

### Enhancer Trap Screen

The X-linked enhancer trap P-element, *PGawB*, was mobilized to generate new insertions as described previously [31]. Briefly, *pGawB/FM7* females were mated to *CyoHop2* males to generate female jumpstarter flies, *pGawB/w-;CyoHop2/+*, which were then mated to the *w-; UAS-eGFP; UAS-eGFP* reporter line. F1 larvae resulting from the jumpstarter-UAS-eGFP reporter cross were flushed from media with water, cleared of debris by floatation on NaCl solution, rinsed in water, and screened for fat body GFP expression under a fluorescence dissecting microsope using a GFP filter. Individual F1 larvae with fat body expression were grown to adults in individual food vials and then mated to the *w-; noc^Sco^/CyO; TM6B.Tb/MKRS* balancer stock and the resulting GFP fluorescing, *CyO*, and *MKRS* F2 progeny were intercrossed to generate lines. Chromosomal assignment for each insertion was achieved by examining segregation of the GFP fluorescence in males and females for X insertions, and by crossing *CyO* and *MKRS* balanced F2 males to *w-; UAS-eGFP; UAS-eGFP* and examining segregation of GFP fluorescence against either the *CyO* or *MKRS* balancer. In the case of X insertions, lines were generated by crossing to *FM7* balancer stocks

### Fat Body Visualization and Nile Red Staining

These studies were done as described [60]. Briefly, larvae or adult flies were submerged in methanol prior to microscopic analysis of the fat body. GFP expression in whole larvae or adults was documented under a fluorescence dissecting scope with a GFP filter. Fat bodies were carefully dissected, fixed and stained with Nile Red and documented under a fluorescence dissecting scope using a rhodamine filter.

### Lifespan Assays

Adults emerging from uncrowded cultures were mated and the resulting larvae were again grown under uncrowded conditions to produce offspring used in lifespan assays. Flies that emerged within a 1–2 day period were pooled and aged for an additional 3 days under standard culture conditions before subjecting to lifespan assays. For the initial screening of the enhancer trap collection in pools of ten lines, which was performed at either 25°C or 30°C, 10 flies per line per sex were combined together in standard food vials and quadruplicate samples were assessed for mortality every other day and then placed into fresh vials. For later lifespan assays with individual lines, ∼80 males and ∼80 females were placed in demography cages in duplicate or triplicate cohorts per trial for lifespan assays performed at room temperature (22–23°C) as described previously [21] and mortality scored daily when changing to fresh food vials.

### Mammalian Adipogenic Cell Culture

3T3-L1 murine preadipocytes were purchased from the American Type Culture Collection and maintained in growth media (DMEM with 10% calf serum, 10 units/ml penicillin, 10 µg/ml streptomycin) at 37°C in 5% CO_2_. Cells were passed before confluence and discarded after 10 passages. Media was changed every other day during cell maintenance and adipogenesis. 3T3-L1 cells were induced to undergo adipogenesis as described [27], [61].

### RNA Extraction, cDNA Synthesis and Real-time PCR

Total RNA from cultured cells or mouse adipose depots were extracted with Trizol (Invitrogen), RNase-free DNAse I-treated, and reverse-transcribed using random hexamers and MMLV-reverse transcriptase (Invitrogen) to obtain cDNA. Gene expression was measured by quantitative real-time PCR analysis using SYBR Green Master Mix reagent (Applied Biosystems, 7500 Real-Time PCR System). Real-time PCR values for gene expression were normalized over endogenous ß-actin expression. All real-time primer sequences were validated for specificity and efficiency prior to use. Real-time primer sequences are available upon request.

### Mouse Studies

Mice were housed in a 12:12 light:dark cycle. For genetic obesity modeling, fat depots were removed from 6 month-old *ob/ob* mice and control littermates fed 4% fat chow (Teklad) [27]. Veterinary care was provided by the Division of Comparative Medicine. All animals were maintained under the guidelines of the U.T. Southwestern Medical Center Animal Care and Use Committee according to NIH guidelines.

### Transgenic Flies

The cDNA clone AT01932 contains the full-length *Snz* (CG1514) cDNA within the pOTB7 vector. Full-length *Snz* cDNA was PCR amplified and cloned into pUAST [32] to generate pUAST-*Snz*. Transgenic lines harboring pUAST-*Snz* were generated using P-element-mediated germline transformation as described previously [62], [63]. Primer sequences used for PCR amplification and sequence verification are available upon request.

### P-element Revertant Screen

Females from the C32 enhancer trap line that contained an insertion in the 5′-UTR of *snz* (CG1514), *snz^C32^*, were crossed to FM7/y;*CyOHop2/+* (transposase source) males to mobilize the P-element. The resulting *snz^C32^*/FM7; *CyoHop2/+* female jumpstarter flies were crossed to *FM7/Y* males. Male white-eyed progeny, lacking the *FM7* balancer, were selected and mated to *pGawB/FM7;+;+* females to generate lines. After mating, genomic DNA was harvested from individual males and PCR amplified with primers flanking the original insertion site to examine the potential P-excision site. *w^1118^* males were used as controls and candidate excision lines were sequenced to assess the excision event. Through this P-excision screen, we obtained multiple lines in which excision at the CG1514 insertion site had occurred, but in all cases a small fragment (25–45 bp) of residual P-element was still present at the excised locus. Revertant lines were maintained without further backcrossing due to the lack of an eye-color marker.

### Oxidative and Heat Stress Assays

Oxidative stress tests were performed as described [64]. Briefly, adult flies emerging on the same day from non-crowded cultures were collected and further cultured in identical conditions on standard fly food for 5 days before stress analyses. For oxidative stress tests, groups of 20 flies per sex per genotype were subject to control or 5% H_2_O_2_ conditions and death was scored every 12 hours. Heat stress assays were performed by placing groups of 20 flies per sex per genotype in a 37°C incubator and scoring for mortality every two hours. Stress tests were performed at least three times and each test was performed in triplicate.

### Negative Geotaxis Assays

Ten flies of the indicated ages were put into empty vials with a line drawn one inch above the bottom. Vials were gently shaken several times and then flies were tapped down to the bottom of the vial. The time required for 50% of flies to migrate one inch above the bottom of the vial was measured. The experiment was performed twice and with triplicate groups of flies per condition.

### Fertility and Fecundity Assays

Flies were grown under standard culture conditions and aged as described in lifespan assays. At various time points five females per genotype were isolated from cultures and introduced to five one week old *w^1118^* males in separate bottles and provided apple juice agar supplemented with yeast paste. After acclimating for 24 hours, flies were given a fresh apple juice plate with yeast paste and allowed to lay eggs for 24 hours. Egg collections were repeated for four 24-hour periods. After each period the total number of eggs and subsequent larvae were counted. Viability was the number of larvae produced divided by the number of eggs laid. Male fertility was assessed in a similar manner using five aged males paired with five one-week old virgin *w^1118^* females. All experiments were performed at least in triplicate.

### *Wolbachia* Detection and Tetracycline Treatment

Female flies were ground with a sterile polypropylene pestle in lysis buffer (10 mM Tris pH 8, 25 mM NaCl, 1 mM EDTA, 200 ug/ml Proteinase K) and incubated for 30 min at 37°C, followed by 10 min at 95°C. 1 µl of lysate was used as template in subsequent PCR assays. PCR detection of *Wolbachia* was carried out as described previously [48]. Fly stocks were reared for two generations in standard fly food containing 0.3 mg/ml tetracycline-HCl (Sigma) to eliminate *Wolbachia* infections.
